# Isolation and characterization of *Leptospira interrogans* serovar Copenhageni from a dog from Saint Kitts

**DOI:** 10.1099/jmmcr.0.005120

**Published:** 2017-10-23

**Authors:** Christopher R. Larson, Michelle Dennis, Rajeev V. Nair, Alejandro Llanes, Andrea Peda, Shamara Welcome, Sreekumari Rajeev

**Affiliations:** ^1^​ Ross University School of Veterinary Medicine, Basseterre, St. Kitts, West Indies; ^2^​ Centro de Biología Celular y Molecular de Enfermedades, Instituto de Investigaciones Científicas y Servicios de Alta Tecnología (INDICASAT AIP), Ciudad del Saber, Panama

**Keywords:** Leptospirosis, Jaundice, hepatocellular dissociation, renal tubular necrosis, interstitial nephritis, antibiotics and supportive therapy

## Abstract

**Introduction.** Leptospirosis is a zoonotic bacterial disease of global distribution affecting humans and animals. The initial phase of leptospirosis resembles many other febrile illness and due to its broad and biphasic clinical manifestations, selection and implementation of appropriate diagnostic tests can be challenging.

**Case presentation.** This report describes a case investigation of a 14 weeks old male, orphan puppy, presented with generalised jaundice, anemia, weakness, and anorexia. Clinical abnormalities included the evidence of renal and hepatic failure. Antemortem and postmortem diagnostic investigations were conducted to identify the cause of illness. PCR testing and culture of blood was positive for *Leptospira* sp. Necropsy followed by histopathology evaluation revealed lesions compatible with liver and kidney damage consisting of marked diffuse hepatocellular dissociation, acute renal tubular necrosis, and mild interstitial nephritis.

**Conclusion.** Multiple diagnostic techniques including bacterial isolation confirmed *Leptospira* infection in this puppy. Whole genome sequencing and analysis identified the *Leptospira* sp. isolated from this puppy as *Leptospira interrogans* serovar Copenhageni. To our knowledge, this case report describes the first isolation of *Leptospira* from Saint Kitts. This case highlights the usefulness of including multiple diagnostic tests for the diagnosis and epidemiological investigation of *Leptospira* infection. Accurate diagnosis followed by timely intervention can prevent case fatality and mortality in infected patients.

## Introduction

Leptospirosis is a zoonotic disease of global importance caused by a spirochete bacteria belonging to the genus *Leptospira* [1]. Pathogenic *Leptospira* infects the renal tubules of a wide variety of domestic and wild animals. Animals may serve as asymptomatic reservoirs or maintenance hosts of *Leptospira*, harboring the bacteria in the renal tubules, and acting as a source of environmental contamination [2]. Other animal hosts and humans, when infected by a non-host adapted, pathogenic *Leptospira,* develop significant disease and are termed as an incidental or accidental host. Leptospirosis has a broad range of clinical manifestations from a mild febrile illness to life-threatening disease presented as hepatic, renal, and pulmonary disease [1–4]. Dogs may act as reservoirs and may succumb to severe illness similar to humans. This report describes a case of *Leptospira* infection in a canine patient and diagnostic investigations leading to the isolation and characterisation of the infecting *Leptospira* species.

## Case Report

A 14 weeks old intact male mixed breed, stray puppy, was presented to Ross University Veterinary Clinic, Saint Kitts in a weak non-ambulatory condition. The patient’s body condition was poor (2/5) and the dehydration status was approximately 12 %. The puppy was covered with brown dog ticks (*Rhipicephalus sanguineus*), and there were several circular areas of hair loss (alopecia). Other notable physical exam findings included icteric mucous membranes and diarrhoea. Temperature, heart rate, and respiratory rate were recorded at 100.3°F, 100 bpm, 32 bpm, respectively. Fluid therapy was initiated immediately through an intravenous catheter.

## Investigations

A SNAP Parvo antigen test (IDEXX laboratories) performed was negative. Complete blood count revealed anemia, thrombocytopenia, and an inflammatory leukogram with a regenerative left shift and toxic changes in neutrophils (Table 1). Azotemia, hyperbilirubinemia, hypoalbuminemia and elevated alkaline phosphatase were detected on the serum biochemistry profile (Table 2). A 4DX SNAP test (IDEXX laboratories) conducted, was positive for both *Ehrlichia* and *Anaplasma*. Therapy was initiated, however, due to the poor prognosis, the puppy was euthanised and the carcass was submitted for postmortem examination.

**Table 1.** Hematology profile

Analyte	Value	Range	Comments
White Blood Cells	35.2×10^9^ cells l^−1^	6−7×10^9^ cells l^−1^	High
Lymphocytes	1.95×10^9^ cells l^−1^	1–4.8×10^9^ cells l^−1^	Normal
Monocytes	0.63×10^9^ cells l^−1^	0.2–1.5×10^9^ cells l^−1^	Normal
Neutrophils	35.27×10^9^ cells l^−1^	3–12×10^9^ cells l^−1^	High
Eosinophils	0.05 cells l^−1^	0–8×10^9^ cells l^−1^	Normal
Basophils	0×10^9^ cells l^−1^	0–2×10^9^ cells l^−1^	Normal
Lymphocytes %	5.50 %	12–30 %	Low
Monocytes %	1.80 %	2–4 %	Low
Neutrophils %	92.50 %	62–87 %	High
Red blood cells	2.73×10^12^ cells l^−1^	5.5–8.5×10^12^ cells l^−1^	Low
Hemoglobin	4.8 g dl^−1^	4.8 g dl^−1^	Low
Hematocrit	15.15 %	37–55 %	Low
Mean Corpuscular volume	56 fL	60–77 fL	Low
Platelets	21×10^9^ cells l^−1^	200–500×10^9^ cells l^−1^	Low

**Table 2.** Serum biochemistry profile

Analyte	Value	Reference range	Comments
ALB	1.7 g dl^−1^	2.5–4.4 g dl^−1^	Low
ALP	256 U l^−1^	20–150 U l^−1^	High
ALT	30 U l^−1^	10–118 U l^−1^	Normal
AMY	620 U l^−1^	200–1200 U l^−1^	Normal
TBIL	4.1 mg dl^−1^	0.1–0.6 mg dl^−1^	High
BUN	49 mg dl^−1^	7–25 mg dl^−1^	High
CA	9.5 mg dl^−1^	8.6–11.8 mg dl^−1^	Normal
PHOS	10.8 mg dl^−1^	2.9–6.6 mg dl^−1^	High
CRE	1.8 mg dl^−1^	.3–1.4 mg dl^−1^	High
GLU	71 mg dl^−1^	60–110 mg dl^−1^	Normal
NA+	137 mg dl^−1^	138–160 mg dl^−1^	Low
K+	4 mmol l^−1^	3.7–5.8 mmol l^−1^	Normal
TP	5.8 g dl^−1^	5.4–8 g dl^−1^	Normal
GLOB	4.1 g dl^−1^	2.3–5.2 g dl^−1^	Normal

**Fig. 3.**
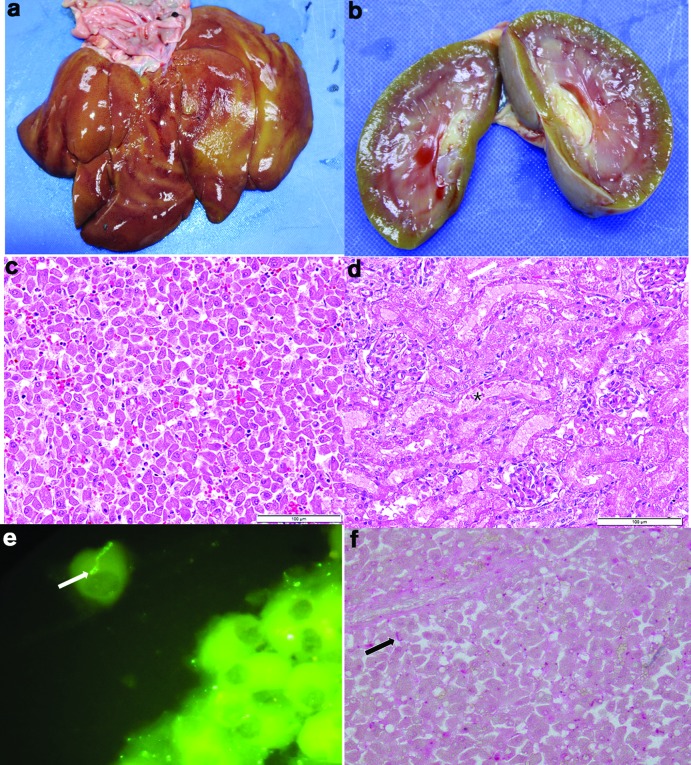
(a) Liver showing diffuse bronze discoloration; (b) Kidney showing slight green yellow discoloration; (c) H and E stain of formalin fixed paraffin embedded section of liver showing hepatocellular dissociation; (d) H and E stain of formalin fixed paraffin embedded section of kidney showing renal tubular necrosis*; (e) Direct fluorescent antibody staining using polyclonal anti-*Leptospira* antibodies conjugated with FITC on the liver. Arrows points to intact spirochetes; (f) Immunohistochemistry of the liver using polyclonal anti-*Leptospira* antibody. Arrows points scattered positive staining for *Leptospira*.

Postmortem findings included severe generalised jaundice, diffuse bronze discoloration of the liver with an accentuated reticular pattern (Fig. 3a), yellow green discoloration of the kidneys (Fig. 3b), and an acute cecocolic intussusception. Histopathological examination revealed marked diffuse hepatocellular dissociation (Fig. 3c), random, mild hepatocellular necrosis and mild cholestasis. Additionally, there was evidence of mild to moderate acute renal tubular epithelial degeneration and necrosis (Fig. 3d). Marked lymphoplasmacytic pancreatitis and necrotising enteritis compatible with parvovirus infection was also present. Direct fluorescent antibody staining (DFA) on fresh urine, and liver (Fig. 3e), and kidney and immunohistochemistry (MACH 4 Universal AP Polymer Kit, Biocare Medical) of the formalin fixed and paraffin embedded liver (Fig. 3f) and kidney sections were pursued using a polyclonal anti-*Leptospira* antibody and were positive for *Leptospira*.

**Fig. 4.**
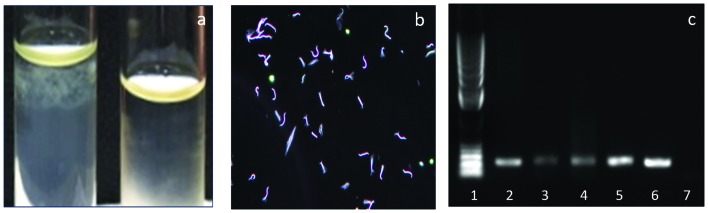
(a) Growth from blood in EMJH semisolid media *Leptospira* is forming subsurface colonies in the tube on the left and no growth in the tube on the right; (b) Image from dark field microscopy of the culture showing spirochetes with morphology compatible with *Leptospira;* (c) Conventional PCR targeting lipL32 gene (Lane 1: DNA Ladder, Lane 2: Blood, Lane 3: Liver, Lane 4: Kidney, Lane 5 and 6: positive controls, Lane 7: negative control).

Microscopic agglutination test (MAT) on the serum was performed against a 20-serovar panel and the results were negative. The Ellinghausen-McCullough-Johnson-Harris (EMJH) media inoculated with blood sample resulted in bacterial growth after a week of incubation. The *Leptospira* growth was observed as subsurface colonies in the semisolid EMJH media (Fig. 4a). Spirochetes compatible with *Leptospira* morphology were observed using dark field microscopy (Fig. 4b). The isolate was confirmed as pathogenic *Leptospira* sp. by conventional PCR (Fig. 4c) and real-time PCR (Fig. S1, available in the online Supplementary Material) targeting the lipL32 gene [5].

To further characterise the *Leptospira* isolate obtained, whole genome sequencing was pursued through next generation sequencing using an Illumina Platform. The phylogenetic analysis was conducted following the methodology discussed in Llanes *et al.* [6]. Maximum likelihood tree was built from the whole genome sequences of 13 *Leptospira interrogans* serovars and the isolate described in this paper. Sequence analysis identified the organism closely related to *L. interrogans* serovar Copenhageni strain Firocruz L1-130. The isolate is designated as *L. interrogans* serovar Copenhageni strain SK1 (Fig. S2).

**Diagnosis:** *Leptospirosis*

**Treatment:** Antibiotics and supportive therapy

**Outcome and follow-up:** Euthanised due to poor prognosis

## Discussion

Leptospirosis is considered as an underdiagnosed and under-reported disease [1]. This case report describes a complete and thorough clinical, diagnostic investigation that has led to the diagnosis of fatal canine leptospirosis and provides an insight into various diagnostic methods that can be applied to suspected cases to reach an accurate diagnosis.

The primary diagnostic methods used in cases of suspected leptospirosis include dark field microscopy (DFM), direct fluorescent antibody test (DFA), PCR, microscopic agglutination test (MAT), and rarely bacterial culture [1]. Variations in sensitivity and specificity are inherent in each of these techniques resulting false positive and false negative results [1]. Bacterial culture and isolation of *Leptospira* are not routinely performed for the diagnosis of leptospirosis, as this procedure is laborious, time consuming and requires special media and expertise [1]. However, the isolation and characterisation of bacteria offers epidemiological information on circulating *Leptospira* species/serovars and its pathogenic attributes. To our knowledge, this report describes the first successful isolation of a *Leptospira* species from the island of Saint Kitts.

Earlier seroprevalence studies conducted in the region (Barbados, Puerto Rico and Trinidad) has shown exposure in dogs to serovars Autumnalis, Icterohaemorrhagiae, Australis, and Pomona [7]. In Puerto Rico, seroprevalence was high in stray dogs to serovar Icterohaemorrhagiae [8]. It is important to note that *Leptospira interrogans* serovar Copenhageni belongs to the serogroup Icterohemorrhagiae which also contains other serovars such as Mankarso and Icterohemorrhagiae and cross agglutination may occur within the members of the serogroup. Serovar Copenhageni has been isolated from the Caribbean islands, Trinidad, and Barbados [9, 10]. In Trinidad, a study in dogs identified 18 % of the suspected cases and 3.4 % of the healthy stray population were infected with the serovar Copenhageni [10].

The identification of this isolate as *Leptospira interrogans* serovar Copenhageni also has broader implications for the region in terms of animal reservoirs and zoonotic potential. This strain is one of the most pathogenic *Leptospira* serovars reported in urban human outbreaks in Brazil and was isolated from 87 % of human leptospirosis cases [11]. Icterohaemorrhagiae was the predominant serogroup in humans and dogs and infections were associated with the infestation of brown rats (*Rattus norvegicus*) in Brazil [11]. The source of infection and transmission in this case, whether environmental or autochthonous is not known. Exposure to a heavily contaminated aquatic environment or contact with other infected animals, such as rodents, or their urine, the common modes of transmission in endemic areas are suspected.

MAT is a serology test widely employed to diagnose leptospirosis and is considered as the gold standard test. This test detects agglutinating antibodies elicited to the infecting *Leptospira* serovar, which do not typically appear in circulation before the first week of infection [1, 12]. Therefore, solely depending on the MAT results can often lead to false negative results in early stages of infection. Single high serum titer (>800) with compatible clinical signs or a four-fold increase in MAT titer on paired sera (acute and convalescent) evaluation are considered as diagnostic for leptospirosis. The usefulness of this test must be considered in light of its limitations. Single titers should be interpreted with caution in endemic areas and in vaccinated populations. Dogs may act as asymptomatic reservoirs of *Leptospira* and this can be a confounding factor in the interpretation of MAT results. A combination of diagnostic tests for the detection of the organism (DFA, PCR) and the antibody response will increase the likelihood of an accurate diagnosis.

The clinical manifestations of leptospirosis in dogs share similarities to human leptospirosis. Two main manifestations of the disease are well described in literature and categorised as both anicteric and icteric forms [1–4]. A biphasic illness may occur in animals consisting of an initial febrile disease due to bacteremia and later, a second immune phase of illness occurs approximately 1–2 weeks post-infection [2–4]. During the second phase, the bacteria reaches its preferred destination, the renal tubules. This may result in renal tubular damage and shedding of the organism in the urine. Both phases of the disease often overlap and are predicated upon the immune status of the host, and the virulence of the infecting *Leptospira* sp. The biphasic nature of the illness and its broad manifestations resembling other febrile illness such as dengue fever pose significant diagnostic challenges in humans [13]. Clinical presentations resembling febrile illness followed by the complications characterised by hepatic and renal abnormalities may be seen in canine patients. Acute forms presented with pulmonary haemorrhage have also been reported in canine patients [14]. Acute hepatocellular dissociation and necrosis, acute renal tubular nephrosis and hemorrhage and chronic interstitial nephritis are some of the salient histopathological features of canine leptospirosis [3, 4]. Icteric disease is reported in association with the serovars Icterohaemorrhagiae and Pomona [15]. Canine leptospirosis cases manifested with only renal pathology are reported in association with infection caused by serovar Grippotyphosa [15, 16].

*Ehrlichia* and *Anaplasma* exposure was confirmed in this patient using SNAP 4Dx test, however, an in-house *Ehrlichia* PCR test was negative. *Anaplasma platys* and *Ehrlichia canis* infections are endemic in Saint Kitts dogs. Similarly, canine parvovirus infection observed on histology also might have contributed to the illness in this puppy. Therefore, the potential occurrence of coinfections should always be considered for effective therapeutic management in patients presented with broad and nonspecific clinical signs. Histopathological evidence of pancreatitis was observed in this patient. There are reports of pancreatitis associated with canine leptospirosis cases [3], but the actual role of *Leptospira* as a cause of pancreatitis warrants further studies. In dogs, severe cases of leptospirosis are manifested as fulminant hepatic and renal dysfunction, resulting in generalised jaundice and/or azotemia. Histologic changes observed in the liver and kidney (diffuse hepatocellular dissociation and interstitial nephritis) were consistent with acute leptospirosis [3, 4].

Leptospirosis is a treatable and to some extent a preventable disease. The members of the antimicrobial group, beta lactams or doxycycline and supportive therapy are the primary choices for treatment in dogs [3, 4]. Canine vaccines are available but the immunity is serogroup specific and vaccines may prevent disease from infections caused by the serovars included in the vaccine preparations lasting about 12 months [3]. Current vaccines in North America contain serovars Icterohaemorrhagiae, Canicola, Grippotyphosa, and Pomona. Bivalent vaccines containing serovars Icterohaemorrhagiae and Canicola are available in Europe [3, 4]. There are more than 250 serovars of *Leptospira* maintained in the renal tubules of reservoir animals and diversity of this varies with geographic regions. This report emphasises the need for active and passive prevalence studies especially in endemic areas using clinical, bacteriological and serological investigations to support clinical decision-making and to develop vaccination strategies/protocols. Geographical, temporospatial, and seasonal factors also play significant roles in the transmission of *Leptospira*. In a recent study we conducted on Saint Kitts, a high overall seroprevalence to *Leptospira* (72.3 %) was observed in dogs (unpublished data). The serovar Autumnalis followed by Icterohemorrhagiae were the predominant serovars in this study. A recent human mortality and morbidity estimate published in a worldwide systematic review found that the Caribbean region has high levels of estimated *Leptospira* related morbidity and mortality rates [17]. Despite the high risk of human leptospirosis in the Caribbean islands including Saint Kitts, animal epidemiological data is sparse in the region. Our future studies will target active surveillance in animals to better understand the transmission dynamics and the potential for using canine population as a sentinel for predicting human *Leptospira* infections.

## Supplementary Data

325 Supplementary File 1
