# Supplementary figures and images for: Isolation and characterization of Leptospira interrogans serovar Copenhageni from a dog from Saint Kitts
Source: JMM Case Rep. 2017 Oct 23;4(10):e005120. doi: 10.1099/jmmcr.0.005120 (PMC5692236; doi:10.1099/jmmcr.0.005120)

Amplification Plot (Rn vs. Cycle)  
**LipL32**

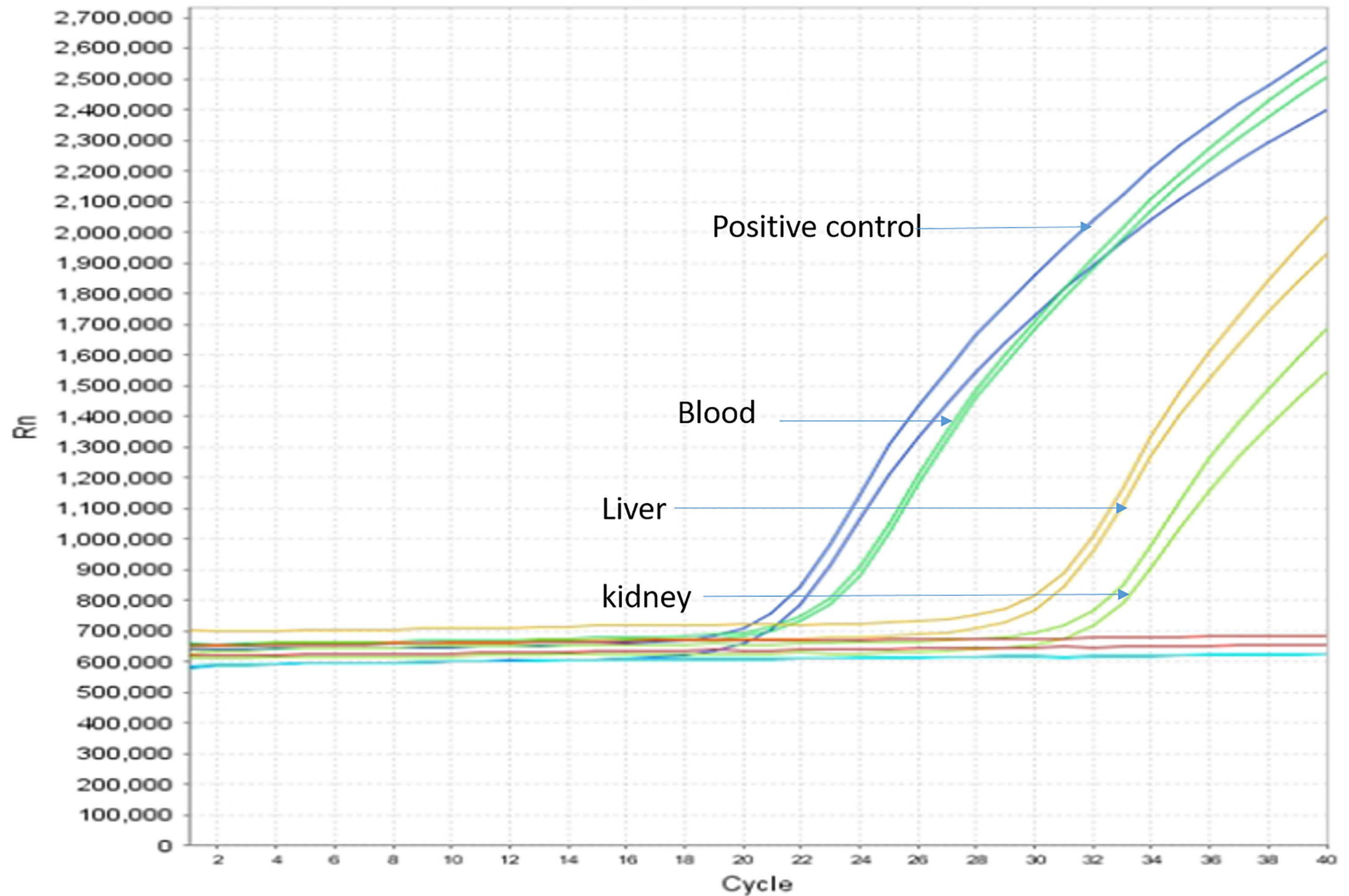

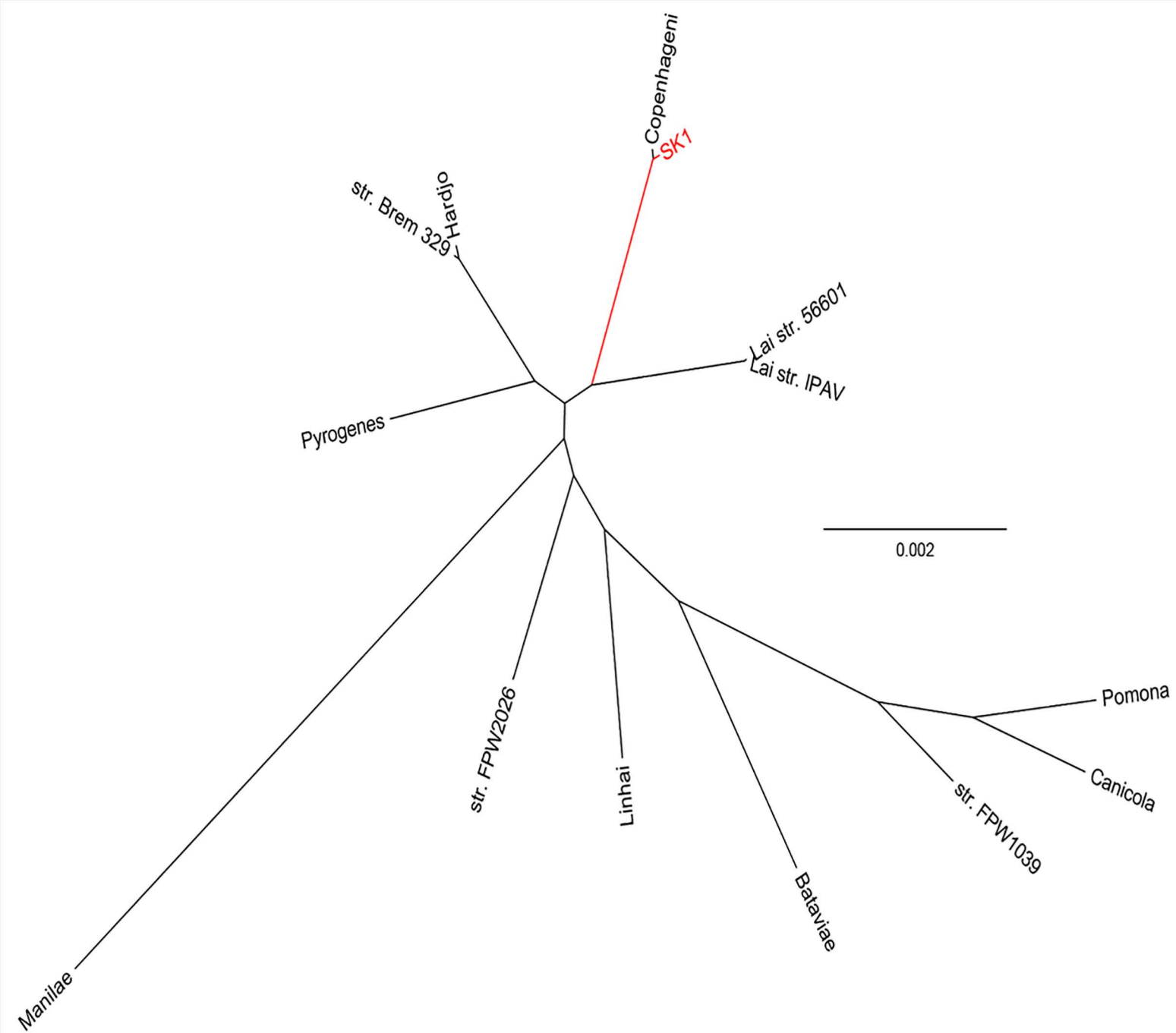

Supplement: Supplementary File 1 [file jmmcr-4-5120-s001.pdf]
